# Distrusting the Process: Electoral Trust, Operational Ideology, and Nonvoting Political Participation in the 2020 American Electorate

**DOI:** 10.1093/poq/nfae025

**Published:** 2024-07-16

**Authors:** Erin B Fitz, Kyle L Saunders

**Affiliations:** PhD Candidate, Department of Political Science, Colorado State University, Fort Collins, CO, US; Professor, Department of Political Science, Colorado State University, Fort Collins, CO, US

## Abstract

This article explores the relationships between electoral trust, operational ideology, and nonvoting political participation (NVP) during the 2020 US presidential election cycle. We hypothesize that: (1) more liberal operational ideology is associated with more NVP, (2) less electoral trust is associated with more NVP, and (3) operational ideology moderates the negative relationship between electoral trust and NVP. Using data from the 2020 American National Election Study (N = 8,280), our contribution is threefold: We first add to previous research that indicated liberals engage in more NVP than conservatives. We then provide some of the first evidence to suggest that electoral trust—in this case, trust prior to the 2020 election—is negatively associated with NVP. Results further indicate that the negative relationship between electoral trust and NVP is strongest among those with conservative operational ideology, such that the more trust those with conservative operational ideology have in the election, the less they engage in NVP. Given that electoral trust is crucial for a well-functioning democracy, the implication is that elites with a strategic incentive to express contempt for the election process can have direct and downstream consequences on political participation.

## Introduction

Scholars in political science have long concluded that elections are the result of short- and long-term forces (Campbell et al. 1960; Converse 1966). While no election is an exception to this idea, the 2020 US presidential election cycle was perhaps the most exceptional: already-increasing elite polarization (McCarty, Poole, and Rosenthal 2006; McCarty 2015), combined with countervailing, myriad concerns specific to this election (e.g., COVID-19, US Supreme Court appointments, and matters of race and inequality; see Pew 2020a), provided multifarious issues from which to elicit ideological convictions and subsequent political participation from the American electorate (Pew 2020b), despite its low trust in government (Pew 2020c).

**Figure 1.**
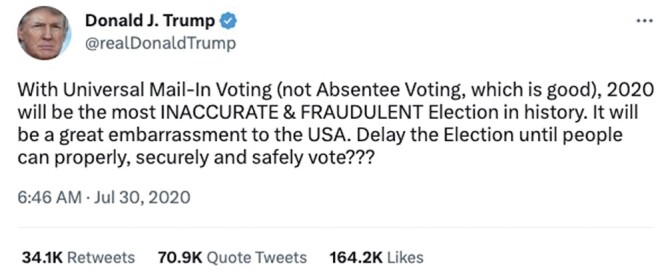
Tweet by @realDonaldTrump on July 30, 2020.

Although it is not uncommon for salient issues to mobilize segments of the electorate (e.g., Saunders and Abramowitz 2004)—nor is it uncommon for political participation to vary despite distrust in government (Hetherington 2006)—the addition of then-president Trump’s rhetoric that sowed skepticism in the integrity of the election (Niedzwiadek 2020; see also figure 1), as well as Democrats’ counternarrative regarding election integrity under Trump (Shear, Fuchs, and Vogel 2020), call into question for whom such ideological cues mattered most. If prior evidence on the causes and consequences of distrust in elections are any indication (e.g., Craig et al. 2006; Alvarez et al. 2013; Norris 2014; Daniller and Mutz 2019; Berlinski et al. 2023), electoral trust should be integral to how certain individuals participate in an election, especially one in which the sitting president sought to denigrate the very system required for his success.

While there has been a great deal of scholarly focus on political and social trust (see, e.g., Hooghe 2017), we know far less about how electoral trust affects the American electorate. We have some evidence, for example, to suggest that there is a positive relationship between electoral trust and voter turnout (Birch 2010), but little knowledge about the relationship between electoral trust and noninstitutionalized, or nonvoting, political participation.^1^ Surveys on the American electorate also tend to only include single-item post-election assessments of election-related attitudes, limiting our ability to reliably understand how these attitudes might affect pre-election beliefs and behaviors (see, e.g., Daniller and Mutz 2019). We argue that examining the relationship between electoral trust and nonvoting participation (NVP) prior to the election is important given that Americans tend to look more toward ideology than partisanship when supporting candidates during this period (Bai 2022) and because ideological cues can motivate individuals to take part in political activities other than voting (Saunders and Abramowitz 2004; Abramowitz 2010; Wojcieszak, Baek, and Delli Carpini 2010). Although NVP captures a variety of political activities associated with, and even shown to mediate (Settle et al. 2016), voting, the latter remains a distinct political activity (Verba and Nie 1972). Thus, a potential implication from the existing participation literature is that elites with a “strategic incentive” (Enders et al. 2021, p. 2) to sow distrust in an election might intend to elicit NVP, and subsequently increase turnout, all the same.

We use data from the 2020 American National Election Study (ANES) to explore the relationships between electoral trust, operational ideology (assessed by individuals’ aggregate issue preferences), and nonvoting political participation (NVP) during the 2020 US presidential election cycle.^2^ Our contribution is threefold: We first add to evidence demonstrating that liberals are more associated with NVP than conservatives. We then provide some of the first evidence to suggest that electoral trust is negatively associated with NVP. We also find that operational ideology moderates the negative relationship between electoral trust and NVP, such that the negative relationship between electoral trust and NVP is strongest among conservatives—that is, the more conservatives trust in the election, the less they engage in NVP.

## Nonvoting Participation

Whereas voting is characterized as an institutionalized act signaling support for one’s partisan team (e.g., Green, Palmquist, and Schickler 2004), NVP encompasses a variety of noninstitutionalized activities, for example, working for or donating to a political party/candidate, protesting, attending political events, wearing or displaying campaign merchandise, contacting public officials, signing petitions, and engaging in politics online (see, e.g., Oser, Hooghe, and Marien 2013; Miller and Saunders 2016). Not only can these activities grant individuals a wider latitude in the type and timing of political participation (see, e.g., van der Meer, van Deth, and Scheepers 2009; Lin and Lunz Trujillo 2023), but they can also affect other political beliefs and behavior (e.g., Settle et al. 2016; Argyle and Pope 2022; Oser et al. 2022). Further, because NVP is associated with, but distinct from, voting (e.g., Verba and Nie 1972), there is potential for the former to serve a more ideological function than the latter (Fitz and Saunders 2023).

There is also some evidence indicating an ideological asymmetry in NVP—specifically, that liberals engage in more NVP than conservatives. One potential reason, according to van der Meer, van Deth, and Scheepers (2009), is that “left-wing preferences aim at changing society, whereas right-wing preferences are about preserving it…As [left-wing citizens] aim to change the societal status quo, they are more likely to participate outside the representational, supposedly elitist, structures” (p. 1431). Another potential explanation is that Democrats represent a broader array of interests, and have been more successful at mobilizing those interests, than Republicans (Grossman and Hopkins 2016). Fitz and Saunders (2023) also found that liberals were more associated with NVP than conservatives during the 2012, 2016, and 2020 US presidential election cycles. Thus, while questions remain as to the mechanism(s) behind this ideological asymmetry, the relationship between liberals and NVP is seemingly distinct neither to the 2020 election, nor to only those elections amidst Republican/conservative presidencies.

Further, while both symbolic and operational ideology are associated with NVP (e.g., Argyle and Pope 2022), and both measures of ideology are strongly correlated with partisan identity, whether and how much individuals take part in NVP often depends on their respective issue positions (e.g., Saunders and Abramowitz 2004; Abramowitz 2010; Wojcieszak, Baek, and Delli Carpini 2010). Combined with evidence demonstrating that issues/operational ideology is a more robust indicator of attitudes compared to one's liberal-to-conservative self-placement (see, e.g., Conover and Feldman 1981; Ellis and Stimson 2012), our primary motivation is to explore the relationship between issues/operational ideology and NVP alongside electoral trust. This is not to suggest that partisan identity and symbolic ideology are not important for political participation; rather, we focus on issues/operational ideology because it has the potential to better capture variation in the motives behind NVP.^3^

## The Role of Electoral Trust

Electoral trust, or one’s confidence in democratic outcomes (see, e.g., Birch 2010), is distinct from political and social trust.^4^ Electoral trust operates independently of the “winning effect” related to political trust (Whiteley et al. 2016), which posits that supporters of the in-party are more satisfied with—and thus more trusting in—the political system to ensure “their” ideal outcome (Singh, Lago, and Blais 2011). Between 2016 and 2020, for example, Republicans became less likely to believe that votes are counted fairly and more likely to trust in government, while Democrats became more likely to believe that votes are counted fairly and less likely to trust in government (ANES 2016, 2020).

But how might electoral trust affect participation? Although we understand that those with greater electoral trust are more likely to vote (Birch 2010), the connection between electoral trust and NVP is less clear, especially given that most prior research examined the relationship between political trust and NVP (Hooghe 2017). Following the logic of previous literature—specifically, the notion that those with less political trust (especially, perhaps, political distrust in certain elites and/or outcomes; see, e.g., Easton 1975) might have a greater propensity for “noninstitutionalized or elite-challenging forms of political participation” (Hooghe 2017, pp 619–20)—we expect to find that those with less electoral trust (at least, those who still wish to participate) would take part in more NVP.

We further expect issues/operational ideology to moderate the relationship between electoral trust and NVP. Although misperceptions of electoral integrity can occur in both parties, top-down messaging plays a key role in constraining partisan beliefs (Enders et al. 2021). In the context of 2020, this suggests Trump’s rhetoric regarding electoral integrity could have helped shape conservatives’ electoral trust (Berlinski et al. 2023), potentially altering whether and to what extent they took part in NVP. Previous evidence indicating that ideological appeals for political change are stronger and more effective for Republicans than Democrats (Grossman and Hopkins 2016), but that those who reject such appeals are less likely to take part in activities beyond voting (Saunders and Abramowitz 2004; Abramowitz 2010), further suggests that the relationship between electoral trust and NVP would be strongest among conservatives. Thus, we hypothesize:

H1: More liberal issues/operational ideology are/is associated with more NVP.

H2: Less electoral trust is associated with more NVP.

H3: Issues/operational ideology moderate(s) the relationship between electoral trust and NVP, such that the negative relationship between electoral trust and NVP is strongest among conservatives.

## Methods

### Data

We tested our hypotheses using the 2020 ANES Time Series Study, which includes two waves (pre- and post-election) of mixed-mode surveys (online, video, and phone) representative of noninstitutional US citizens aged 18 or older. Pre-election interviews (N = 8,280) were conducted between August 18, 2020, and November 3, 2020; post-election re-interviews (N = 7,449) were conducted between November 8, 2020, and January 4, 2021.^5^

### Dependent Variable

*NVP* is an additive scale of 12 binary self-reported measures of political participation. The scale consists of activities included in the ANES over time (i.e., talking to others about voting, attending political events, working for a party or candidate, wearing or displaying a campaign button/sticker/sign, and donating money to a party or candidate; see, e.g., Saunders and Abramowitz 2004; Abramowitz 2010; Lupton, Myers, and Thornton 2015) and others included in more recent ANES surveys (i.e., online participation, contacting a member of Congress, protesting, signing a petition, and donating to a political group). As emphasized by previous scholarship, accounting for a broader array of activities helps us better capture one's general propensity to participate (Verba and Nie 1972), especially given that much has changed with regard to how people can participate since the ANES began asking about these activities more than sixty years ago (Fitz and Saunders 2023). Thus, including more contemporary modes of NVP with those assessed over time, as we can do with these data from 2020, results in (what we argue is) a more temporally and conceptually valid indicator of *NVP*.

In the post-election portion of the survey, the 2020 ANES asked respondents whether they, during the campaign/in the last 12 months: (1) talked to people about voting, (2) participated in online political events in support of a particular candidate, (3) went to political events in support of a particular candidate, (4) wore or displayed a campaign button/sticker/sign, (5) worked for a party or candidate, (6) gave money to a candidate, (7) gave money to a party, (8) gave money to a group that supported or opposed candidates, (9) joined a protest, march, rally, or demonstration, (10) signed an online or paper petition about a political or social issue, (11) posted a message or comment online about a political issue or campaign, and (12) contacted or tried to contact a member of Congress. Despite the range in frequency at which respondents self-reported these activities (shown in parentheses next to each activity listed in table 1), roughly 70 percent of respondents self-reported at least one activity (*M *=* *2.14, *SD *=* *2.31). Item and factor analyses (also in table 1), as well as robustness checks that further assess individual activities included in *NVP* (see Supplementary Material SM1), confirm that these items fit together well into a unidimensional scale (α = 0.77).

**Table 1.** Item and factor analyses.

	NVP	Electoral Trust		Issues
Talk to others (0.42)	0.40			
Comment online (0.39)	0.44			
Sign online/paper petition (0.28)	0.51			
Contribute money to candidate (0.20)	0.72			
Wear or Display button/sign/sticker (0.17)	0.42			
Contact Senate/house member (0.17)	0.49			
Attend online meeting/rally (0.13)	0.65			
Contribute money to party (0.13)	0.60			
Join a protest (0.09)	0.42			
Contribute money to political group (0.06)	0.45			
Attend meeting/rally (0.05)	0.44			
Work for party/candidate (0.04)	0.38			
				
Trust in government		0.29		
Trust in people		0.31		
Trust in election officials		0.82	0.77	
Votes counted accurately		0.74	0.77	
				
Government vs. private health insurance				0.76
Guaranteed jobs and income				0.76
Aid to Black people				0.74
Government services/spending				0.70
Environment/business trade-off				0.74
Border wall with Mexico				0.77
Defense spending				0.56
				
Eigenvalue	3.06	1.40	1.19	3.66
Proportion of shared variance	0.57	0.81	1.00	0.85
Cronbach’s Alpha	0.77	0.60	0.74	0.87
*N*	7,430	8,184	8,211	8,180

### Independent Variables

*Electoral Trust* combines responses to two questions from the pre-election portion of the survey: “How much do you trust the officials who oversee elections where you live?” (with response options on a five-point scale ranging from “not at all” to “a great deal”) and “In the November 2020 general election, how accurately do you think the votes will be counted?” (with response options on a five-point scale ranging from “not at all accurately” to “completely accurately.”)^6^ Item and factor analyses indicate these items fit together well (α = 0.74) and that trust in government and trust in people do not measure the same latent construct (see table 1).^7^

*Issues*, or operational ideology, averages together respondents’ self-placement on seven seven-point questions on the following issues, included in the pre-election survey: (1) government vs. private health insurance, (2) guaranteed jobs and income, (3) aid to Black people, (4) government services/spending, (5) environment/business trade-off, (6) the border wall with Mexico, and (7) defense spending (α = 0.89, see table 1). Prior to averaging these items together, we recoded all responses for “haven’t thought much about this” as four and reverse-coded responses to the border wall question such that all items directionally correspond with partisan identity and symbolic ideology.^8^ As expected (see, e.g., Conover and Feldman 1981), *Issues* is strongly correlated with partisan identity and symbolic ideology (*r *=* *0.72 and *r *=* *0.75, respectively).

We also control for whether respondents were *Recruited* to participate, attitudes on prospective *Finances*, *Trust in Government, Trust in People, Party ID Strength*, *Ideological Strength*, *Efficacy*, *Interest*, *Anger*, *Sex, Education*, *Religiosity*, *Race*, *Ethnicity*, and *Income*. We recoded all variables in our analysis to range from 0 to 1.^9^

## Results

We show results from the set of Poisson regression models for *NVP* in table 2. In line with *H1* and *H2*, results for Model 1 show that more liberal *Issues* (as indicated by the negative coefficient) is associated with more *NVP*; less *Electoral Trust* is also associated with more *NVP*. Results for Model 2 support *H3* by indicating a negative relationship between the interaction term for *Electoral Trust* x *Issues* and *NVP*.

**Table 2.** NVP in 2020. B denotes Poisson regression coefficients; SE denotes standard errors.

	(1)			(2)		
	B	SE	*p*-value	B	SE	*p*-value
Issues	−0.36	(0.07)	0.000	0.03	(0.15)	0.818
Electoral trust	−0.21	(0.08)	0.013	0.12	(0.11)	0.298
Electoral trust x issues				−0.80	(0.24)	0.002
Trust in government	−0.01	(0.07)	0.885	−0.01	(0.07)	0.857
Trust in people	0.29	(0.07)	0.000	0.28	(0.08)	0.001
Party ID strength	0.14	(0.05)	0.013	0.13	(0.05)	0.016
Ideological strength	0.63	(0.05)	0.000	0.63	(0.05)	0.000
Interest	0.92	(0.05)	0.000	0.91	(0.05)	0.000
Education	0.27	(0.06)	0.000	0.27	(0.06)	0.000
Income	0.18	(0.05)	0.001	0.17	(0.05)	0.003
Religiosity	0.01	(0.05)	0.805	0.02	(0.05)	0.740
Age	−0.10	(0.06)	0.089	−0.11	(0.06)	0.076
Sex	0.05	(0.04)	0.202	0.05	(0.04)	0.192
Race	0.12	(0.05)	0.011	0.11	(0.05)	0.021
Ethnicity	0.00	(0.07)	0.977	0.00	(0.07)	0.985
Recruited	0.62	(0.04)	0.000	0.62	(0.04)	0.000
Efficacy	0.09	(0.06)	0.170	0.08	(0.06)	0.170
Anger	0.48	(0.06)	0.000	0.46	(0.06)	0.000
Finances	−0.07	(0.09)	0.445	−0.08	(0.09)	0.393
Constant	−3.60	(0.11)	0.000	−3.72	(0.11)	0.000
*N*	6,794			6,794		

**Figure 2.**
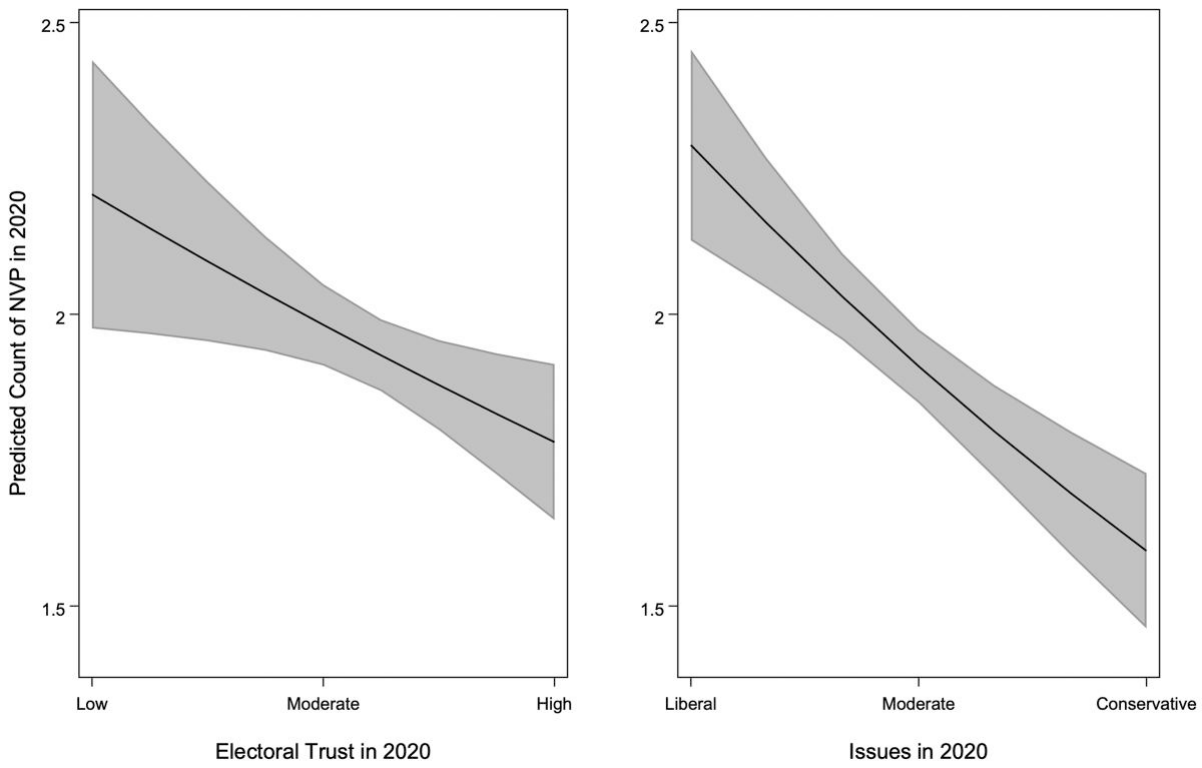
Predicted effects of electoral trust and issues on NVP in 2020. Figures correspond with results shown in table 2, Model 1.

We illustrate the predicted marginal effects for *Electoral Trust* and *Issues* on *NVP* in 2020 (based on results from table 2, Model 1) in figure 2. The average predicted count of *NVP* is highest for those with the least *Electoral Trust* (2.21) and lowest for those with the most *Electoral Trust* (1.78). The average predicted count of *NVP* is also highest for those with the most liberal *Issues* (2.29) and lowest for those with the most conservative *Issues* (1.59).

**Figure 3.**
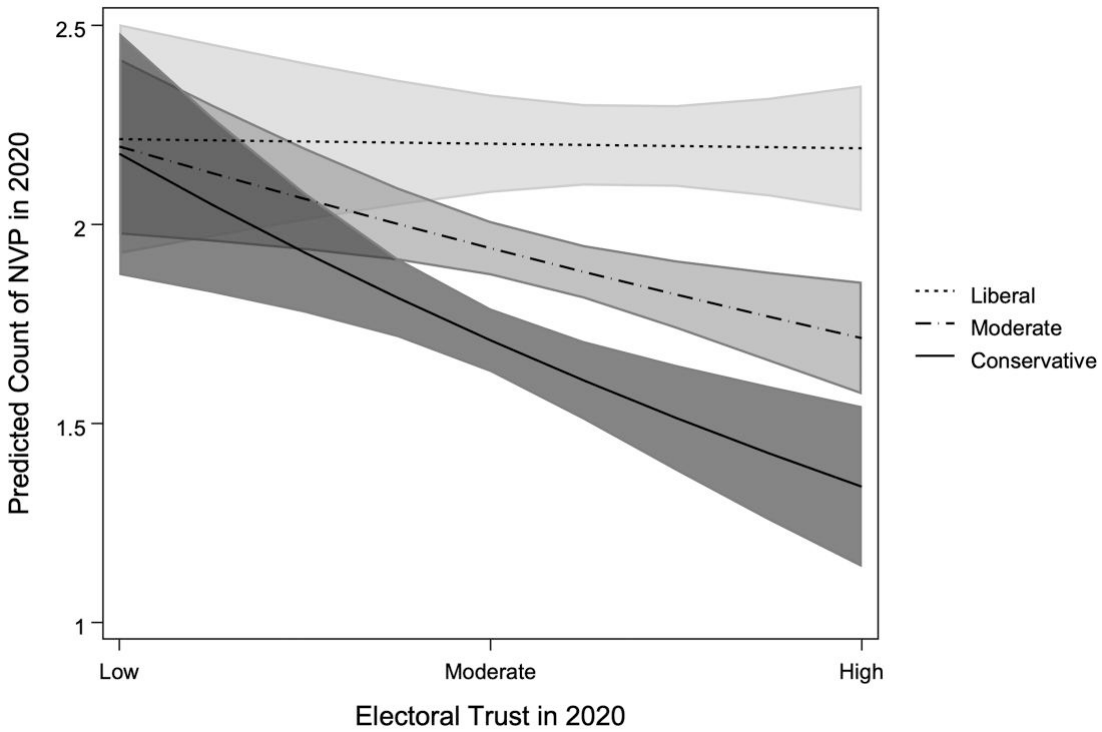
Predicted effect of electoral trust x issues on NVP in 2020. Figure corresponds with results shown in models using binned Issues measure (see Supplementary Material SM5).

To better interpret results from the interaction term in Model 2, we created an additional independent variable that bins *Issues* into thirds, yielding approximate groupings of liberal, moderate, and conservative *Issues*. Results in models using this binned measure of *Issues* (see Supplementary Material SM5) are not substantively different from those shown in table 2. Using this binned measure to calculate predicted marginal effects, we find that the predicted counts of *NVP* at observed values of *Electoral Trust* (illustrated in figure 3) range from 2.21 to 2.19 for liberals, 2.20 to 1.71 for moderates, and 2.18 to 1.34 for conservatives. Thus, in line with *H3*, these results suggest that the negative relationship between *Electoral Trust* and *NVP* is strongest among conservatives.

## Discussion and Conclusion

Having asked how electoral trust and issues/operational ideology affected NVP during the 2020 US presidential election cycle, our findings reveal a positive relationship between more liberal *Issues* and *NVP* (*H1*), as well as a negative relationship between *Electoral Trust* and *NVP* (*H2*). We also find that *Issues* moderates the relationship between *Electoral Trust* and *NVP*, such that the negative relationship between *Electoral Trust* and *NVP* is strongest among conservatives (*H3*). Our findings are robust to a variety of other factors—including other types of political and social trust often discussed, but rarely accounted for together, in the extant literature.

Still, we cannot assess causality with these data or rule out potential measurement error (see, e.g., Ansolabehere and Hersh 2012); we also cannot yet, given the available data, assess the generalizability of these findings in other election cycles. Our findings are, however, robust to tests substituting alternative measures of *NVP*, providing further evidence to suggest that the relationships observed with the present data are not contingent upon specific activities, but rather one’s general propensity to engage in NVP (see again Supplementary Material SM1). Similarly, our findings are robust to tests substituting alternative measures of *Issues*, suggesting that the relationships observed with the present data are not contingent upon specific issues, but rather one’s general operational ideology (see Supplementary Material SM6). Thus, we look forward to work that can assess the robustness of our findings over time.

Our findings, along with prior research on reactions to winning/losing across multiple election cycles (Daniller and Mutz 2019) and the performance of election-denying candidates in the 2022 US midterm elections (Malzahn and Hall 2023), also call into question the incentive(s) for elites to facilitate or obstruct electoral trust. Future research might explore, for example, whether there is a feedback loop between NVP and electoral trust, or whether electoral trust is contingent on, or even a proxy for, trust in one’s party or party leaders. Finally, we encourage scholars to examine whether certain individuals resort to NVP to purposely mobilize outside the institution(s) they distrust, especially given recent research demonstrating that some individuals act not in defense of their chosen partisan side, but “to tear down the established system” (Petersen, Osmundsen, and Arceneaux 2023, p. 4; see also Farhart et al. 2023).

To conclude, our finding that greater electoral trust decreased NVP in 2020 may imply that sowing distrust in the election process is a useful political tool. What we cannot affirm with these data, however, is whether these effects are the direct result of Trump’s messaging regarding electoral integrity, conservatives’ propensity to make ideological appeals, or whether it is rather, as Enders et al. (2021) described, “completely dependent on circumstances, especially the willingness of partisan elites to allege fraud” (p. 7; see also Valentino and Neuner 2017). Regardless, our results help further signal the potential downstream effects of when individuals reject dubious claims about the election process, for example, diminished candidate and party support, the decreased likelihood they will participate (and thus mobilize others to do so), and even whether they turn out to vote. As such, the larger takeaway from this research is that expressing unsubstantiated contempt for the election process—whether to elicit participation from supporters or as a result thereof—might influence the electorate but is not necessarily without consequence.

## Supplementary Material

nfae025_Supplementary_Data

## Data Availability

Replication data and documentation are available at https://doi.org/10.7910/DVN/BVIMHX.

## Footnotes

1 Our conceptualization of nonvoting participation (NVP) is not meant to refer to those who do not vote; rather, NVP refers only to those political activities that are not voting.

2 As we later discuss, operational ideology is a scaled average of issue positions; issues/operational ideology is also strongly correlated with (and directionally corresponds to) symbolic ideology, that is, one’s liberal to conservative self-placement. Thus, for brevity, we use the terms “issues” and “operational ideology” interchangeably throughout this manuscript, and any reference we make to a specific ideology (i.e., liberal, moderate, or conservative) refers to individuals’ aggregate issues/operational ideology.

3
Fitz and Saunders (2023) used multiple measures of partisanship and ideology to explore NVP in 2020 and consistently found that ideology, as opposed to partisanship, is associated with NVP.

4 Broadly conceived, political trust is “the degree to which people perceive that government is producing outcomes consistent with their expectations” (Hetherington 2006, p. 9). Interpersonal trust is “the willingness to accept vulnerability based upon positive expectations about another’s behavior” (Dunn and Schweitzer 2005, p. 736).

5 The 2020 ANES includes reinterviews with 2016 respondents and a fresh cross-sectional sample. The pre-election response rate was 77.9 percent for 2016 reinterviews and 40.9 percent for the cross-sectional sample; the post-election response rate was 90 percent overall. All models in our analysis include survey weights for the full sample.

6 While some might argue that a scaled average of *Electoral Trust* stacks the odds against other covariates, results from models wherein we substituted individual items for *Electoral Trust* (see Supplementary Material SM2) suggest that the latter is a robust, but more conservative, measure of election-related attitudes.

7 As shown in table 1, combining all measures of trust reduces Cronbach’s Alpha from 0.74 to 0.60. Factor loadings for trust in government and trust in people are also at or below the accepted threshold of 0.30.

8 Results from models in which we omitted all responses for “haven’t thought much about this” were not substantively different from those shown herein.

9 Full question wording and summary statistics for all variables in our analysis are in Supplementary Material SM3 and SM4.
