# Supplementary material for: Distrusting the Process: Electoral Trust, Operational Ideology, and Nonvoting Political Participation in the 2020 American Electorate
Source: Public Opin Q. 2024 Jul 16;88(SI):843–57. doi: 10.1093/poq/nfae025 (PMC11300041; doi:10.1093/poq/nfae025)
Supplement: nfae025_Supplementary_Data [file nfae025_supplementary_data.pdf]

Supplementary Material for: Distrusting the Process: Electoral Trust, Operational Ideology, and Non-Voting Political Participation in the 2020 American Electorate

Erin B. Fitz[1] and Kyle L. Saunders[2]

[1]Department of Political Science, Colorado State University
erin.fitz@colostate.edu, ORCiD 0000-0002-0780-0524
[2]Department of Political Science, Colorado State University
kyle.saunders@colostate.edu, ORCiD 0000-0003-4023-8729

SM1: Robustness Checks with Alternative Measures of *NVP* 1

SM2: Individual Measures in *Electoral Trust* 8

SM3: Question Wording 9

SM4: Summary Statistics 17

SM5: NVP in 2020 (with Binned *Issues*) 18

SM6: Alternative 2020 *Issues* 19

SM1: Robustness Checks with Alternative Measures of *NVP*

While our aim is to build upon prior research that assessed a broad array of non-voting activities in 2020, it remains important to emphasize that there is no consensus as to a systematic study of political participation. There is, however, a body of extant literature that used a six-item measure of *NVP* from the ANES–notably, also often considering the six activities together as a meaningful, unidimensional scale (e.g., Abramowitz 2010; Saunders and Abramowitz 2004; Lupton, Myers, and Thornton 2015). Since 1952, the ANES has consistently assessed whether respondents, during or prior to a given election: 1) attended a meeting/rally, 2) wore/displayed a campaign button/sign/sticker, 3) donated to a political party, 4) donated to a candidate, 5) worked for a party/candidate, and 6) talked to others about voting.[1] In turn, although research using the six-item *NVP* is perhaps the closest we have to a systematic study of participation, it is arguably more so an artifact of these questions being included in the ANES over time than it is any consensus that these are the only substantive activities other than voting.

Still, we aim to speak to this extant literature by conducting an additional set of robustness checks that substitute the six-item *NVP* for the 12-item *NVP* used in our main analysis. We do so by creating an additional measure for the six-item *NVP* that includes the activities described above and find–as would be expected–that these activities fit together well into a unidimensional scale ($\alpha = 0.62$).

We show results from this set of Poisson regression models for the six-item *NVP* in

[1]Prior to 1988, the ANES included one question that assessed whether respondents had donated to a political party or candidate. ANES surveys fielded from 1988 forward included two questions, one assessing whether they had donated to an individual candidate and another assessing whether they had donated to a political party. As such, various literature using these items refers to them as both five- and six-item measures of non-voting campaign activity.

Table SM1.1. Results for Model 1 are somewhat contrary to findings from our main analysis in that neither *Issues* or *Electoral Trust* are statistically significant. The interaction term for *Electoral Trust* x *Issues* is statistically significant and negative, however, providing more evidence to suggest that there is a conditional relationship between *Issues* and *Electoral Trust* on *NVP* (*H3*).

	(1)			(2)		
	B	SE	p-value	B	SE	p-value
Issues	-0.14	(0.09)	0.129	0.25	(0.16)	0.133
Electoral Trust	-0.12	(0.10)	0.240	0.22	(0.16)	0.171
Electoral Trust x Issues				-0.75	(0.26)	0.006
Trust in Government	0.15	(0.10)	0.135	0.15	(0.10)	0.131
Trust in People	0.21	(0.09)	0.031	0.20	(0.09)	0.040
Party ID Strength	0.36	(0.06)	0.000	0.35	(0.06)	0.000
Ideological Strength	0.54	(0.05)	0.000	0.53	(0.05)	0.000
Interest	1.08	(0.07)	0.000	1.07	(0.07)	0.000
Education	0.16	(0.07)	0.024	0.16	(0.07)	0.026
Income	0.20	(0.06)	0.001	0.19	(0.06)	0.002
Religiosity	0.11	(0.06)	0.072	0.12	(0.06)	0.061
Age	0.19	(0.07)	0.005	0.19	(0.06)	0.005
Sex	-0.09	(0.04)	0.022	-0.09	(0.04)	0.022
Race	0.13	(0.05)	0.016	0.12	(0.05)	0.026
Ethnicity	0.02	(0.08)	0.820	0.02	(0.08)	0.840
Recruited	0.68	(0.04)	0.000	0.68	(0.04)	0.000
Efficacy	0.12	(0.07)	0.100	0.12	(0.07)	0.098
Anger	0.43	(0.07)	0.000	0.41	(0.08)	0.000
Finances	0.10	(0.11)	0.363	0.09	(0.11)	0.395
Constant	-4.23	(0.12)	0.000	-4.37	(0.12)	0.000
N		6794			6794	

Table SM1.1. Six-Item *NVP* in 2020. B denotes Poisson regression coefficients; SE denotes standard errors.

Given these mixed results, we followed the course of previous participation scholarship to further assess the structure of all activities included in the 12-item *NVP* (Verba and Nie 1972) and examine whether any specific activity in the 12-item *NVP* is driving our main results (e.g., Argyle and Pope 2022).

To assess the former, we take a step back from the item and factor analyses discussed in the main analysis to examine correlation coefficients between individual activities in the 12-item *NVP*. While we echo others who emphasized that examining the structures of these measures according to their frequencies and correlations can inadvertently mask overlapping distributions (Verba and Nie 1972), we acknowledge that assessing frequencies (as shown in Table 1 of the main analysis) and correlations (as described below) remains a useful exercise in understanding how these items are interrelated and thus whether they are appropriate to model as fewer (or, in our case, one) latent factors.

To assess the latter, we created an additional variable, *Any NVP*, coded 0 for those who self-reported no *NVP* and 1 for those who self-reported any non-zero count of *NVP*. We present the pairwise correlation coefficients between individual items in the 12-item *NVP* in Table SM1.2. We also present the pairwise correlation coefficients between *Any NVP* and these individual items in the last column of Table SM1.2.

	Attend Online	Attend	Wear	Work	Contribute: Candidate	Contribute: Party	Contribute: Group	Protest	Petition	Comment	Contact	Talk	Any NVP
Attend Online	-	0.40	0.28	0.28	0.45	0.34	0.27	0.25	0.27	0.26	0.29	0.23	0.26
Attend	0.40	-	0.25	0.24	0.20	0.14	0.10	0.29	0.13	0.15	0.14	0.13	0.16
Wear	0.28	0.25	-	0.14	0.25	0.21	0.14	0.19	0.20	0.23	0.18	0.24	0.30
Work	0.28	0.24	0.14	-	0.23	0.18	0.21	0.16	0.14	0.11	0.18	0.15	0.13
Contribute: Candidate	0.45	0.20	0.25	0.23	-	0.67	0.34	0.18	0.24	0.20	0.30	0.22	0.33
Contribute: Party	0.34	0.14	0.21	0.18	0.67	-	0.28	0.11	0.20	0.13	0.23	0.17	0.25
Contribute: Group	0.27	0.10	0.14	0.21	0.34	0.28	-	0.19	0.21	0.15	0.27	0.15	0.17
Protest	0.25	0.29	0.19	0.16	0.18	0.11	0.19	-	0.29	0.20	0.23	0.15	0.21
Petition	0.27	0.13	0.20	0.14	0.24	0.20	0.21	0.29	-	0.36	0.36	0.23	0.42
Comment	0.26	0.15	0.23	0.11	0.20	0.13	0.15	0.20	0.36	-	0.25	0.28	0.52
Contact	0.29	0.14	0.18	0.18	0.30	0.23	0.27	0.23	0.36	0.25	-	0.21	0.30
Talk	0.23	0.13	0.24	0.15	0.22	0.17	0.15	0.15	0.23	0.28	0.21	-	0.56
N	7449	7447	7447	7447	7449	7445	7442	7446	7447	7445	7446	7448	7449
M	0.30	0.20	0.21	0.18	0.30	0.24	0.21	0.20	0.24	0.21	0.24	0.20	0.30

Table SM1.2: Pairwise Correlations for Individual NVP Activities and *Any NVP*. Table values are pairwise correlation coefficients for unweighted covariates. All correlation coefficients are statistically significant at $p < 0.05$.

Looking first to the correlations between each activity in the 12-item *NVP*, shown in the first 12 columns of Table SM1.2, the mean correlation coefficient between each activity and all other activities (shown in the bottom row–denoted by M) ranges between 0.18 and 0.30; the mean of all correlation coefficients across all cells in the correlation table (excluding those for *Any NVP*) is 0.23. All correlation coefficients are statistically significant and positive. Altogether, these results mirror those from previous scholarship (e.g., Verba and Nie 1972) by indicating that all individual activities in the 12-item *NVP* are neither separate nor uncorrelated; thus, engaging in any one mode of NVP increases the probability of engaging in any other mode of NVP. Moreover, these results bolster the item and factor analyses in our main analysis by further demonstrating that these 12 activities can be combined into a meaningful, unidimensional measure of *NVP* wherein "citizens have general propensities to be active or passive" (Verba and Nie 1972, 59).

Looking to correlations between these individual items and *Any NVP*, the mean correlation coefficient (shown in the bottom row under *Any NVP*) is 0.30. Here again, all correlation coefficients are statistically significant and positive, further indicating that engaging in one mode of NVP increases the probability of engaging in any other mode of NVP. With regard to those activities not included in the six-item *NVP* (but included in the 12-item *NVP*), *Comment* (i.e., commenting about politics online) is most strongly correlated with *Any NVP* ($r = 0.52$). Thus, we created an additional independent variable that adds *Comment* to the six-item *NVP*. Results from models wherein we substituted this seven-item measure, shown in Table SM1.3, indicate that *Electoral Trust* is statistically significant and negatively associated with NVP; *Issues*, however, remains non-significant.

Considering the mean NVP in our sample (2.14), results from item and factor analyses, as well as the mean correlation between *Comment* and the other 11 activities in the 12-item *NVP* (0.21), we take this to suggest that the 12-item *NVP* provides more variance from which *Issues* can have a stronger association with *NVP*. Further, we conclude it is the varied combinations of *NVP* activities, rather than any one activity, driving the signif-

icant results for *Electoral Trust* and *Issues* on *NVP* in our main analysis. This interpretation aligns with previous scholarship that found certain individuals might emphasize, but typically do not exclusively engage in, a single mode of political participation over another (Oser, Hooghe and Marien 2013).

Thus, while it may be the case that a greater proportion of individuals reported commenting about politics online (compared to most other activities), and those who commented online had a higher probability of engaging in other activities, these results do not suggest that *Comment* is the driving force behind our main results–and, to that end, neither is *Talk*, which has the greatest proportion of all activities and strongest correlation with *Any NVP*, and notably *is* part of the six-item measure of *NVP* (for which *Issues* and *Electoral Trust* were non-significant).

	(1)			(2)		
	B	SE	p-value	B	SE	p-value
Issues	-0.15	(0.08)	0.058	0.19	(0.15)	0.196
Electoral Trust	-0.19	(0.09)	0.042	0.11	(0.13)	0.383
Electoral Trust x Issues				-0.68	(0.23)	0.005
Trust in Government	0.10	(0.08)	0.217	0.10	(0.08)	0.218
Trust in People	0.23	(0.08)	0.008	0.22	(0.08)	0.012
Party ID Strength	0.24	(0.05)	0.000	0.23	(0.05)	0.000
Ideological Strength	0.53	(0.05)	0.000	0.52	(0.05)	0.000
Interest	0.90	(0.06)	0.000	0.89	(0.06)	0.000
Education	0.14	(0.06)	0.020	0.14	(0.06)	0.022
Income	0.14	(0.05)	0.010	0.12	(0.05)	0.020
Religiosity	0.04	(0.05)	0.388	0.05	(0.05)	0.347
Age	0.03	(0.06)	0.572	0.03	(0.06)	0.608
Sex	-0.01	(0.03)	0.771	-0.01	(0.03)	0.786
Race	0.13	(0.05)	0.005	0.13	(0.05)	0.009
Ethnicity	0.03	(0.07)	0.681	0.03	(0.07)	0.702
Recruited	0.59	(0.04)	0.000	0.59	(0.04)	0.000
Efficacy	0.07	(0.06)	0.249	0.07	(0.06)	0.248
Anger	0.46	(0.06)	0.000	0.44	(0.06)	0.000
Finances	-0.03	(0.09)	0.721	-0.04	(0.09)	0.667
Constant	-3.54	(0.11)	0.000	-3.65	(0.11)	0.000
N		6794			6794	

Table SM1.3: Seven-Item *NVP* (Six-Item *NVP* with *Comment*). B denotes Poisson regression coefficients; SE denotes standard errors.

SM2: Individual Measures in *Electoral Trust*

	(1)			(2)			(3)			(4)		
	B	SE	p-value	B	SE	p-value	B	SE	p-value	B	SE	p-value
Issues	-0.33	(0.07)	0.000	-0.03	(0.14)	0.855	-0.37	(0.07)	0.000	-0.09	(0.12)	0.473
Trust Election Officials	-0.12	(0.07)	0.095	0.12	(0.11)	0.261						
Trust Election Officials x Issues				-0.55	(0.19)	0.006						
Vote Count							-0.20	(0.07)	0.004	0.06	(0.09)	0.505
Vote Count x Issues										-0.64	(0.22)	0.005
Trust in Government	-0.03	(0.07)	0.670	-0.04	(0.07)	0.579	-0.02	(0.07)	0.833	-0.01	(0.07)	0.884
Trust in People	0.29	(0.08)	0.000	0.27	(0.08)	0.001	0.28	(0.08)	0.001	0.27	(0.08)	0.001
Party ID Strength	0.13	(0.05)	0.015	0.13	(0.05)	0.017	0.13	(0.05)	0.015	0.13	(0.05)	0.018
Ideological Strength	0.64	(0.05)	0.000	0.64	(0.05)	0.000	0.63	(0.05)	0.000	0.62	(0.05)	0.000
Interest	0.92	(0.05)	0.000	0.91	(0.05)	0.000	0.92	(0.05)	0.000	0.91	(0.05)	0.000
Education	0.27	(0.06)	0.000	0.26	(0.06)	0.000	0.27	(0.06)	0.000	0.27	(0.06)	0.000
Income	0.18	(0.05)	0.002	0.16	(0.05)	0.004	0.18	(0.05)	0.002	0.16	(0.05)	0.003
Religiosity	0.01	(0.05)	0.780	0.02	(0.05)	0.696	0.01	(0.05)	0.785	0.01	(0.05)	0.784
Age	-0.12	(0.06)	0.055	-0.12	(0.06)	0.043	-0.11	(0.06)	0.063	-0.11	(0.06)	0.059
Sex	0.05	(0.04)	0.158	0.05	(0.04)	0.162	0.05	(0.03)	0.188	0.05	(0.03)	0.162
Race	0.12	(0.05)	0.013	0.11	(0.05)	0.020	0.12	(0.05)	0.012	0.11	(0.05)	0.021
Ethnicity	0.00	(0.07)	0.969	-0.00	(0.07)	0.989	0.00	(0.07)	0.979	0.00	(0.07)	0.993
Recruited	0.62	(0.04)	0.000	0.62	(0.04)	0.000	0.62	(0.04)	0.000	0.62	(0.04)	0.000
Efficacy	0.08	(0.06)	0.239	0.07	(0.06)	0.262	0.08	(0.06)	0.195	0.08	(0.06)	0.186
Anger	0.48	(0.06)	0.000	0.47	(0.06)	0.000	0.47	(0.06)	0.000	0.46	(0.06)	0.000
Finances	-0.07	(0.09)	0.470	-0.08	(0.09)	0.418	-0.08	(0.09)	0.392	-0.08	(0.09)	0.361
Constant	-3.64	(0.11)	0.000	-3.73	(0.11)	0.000	-3.58	(0.11)	0.000	-3.67	(0.10)	0.000
N	6790			6790			6780			6780		

Table SM2: Individual Measures in *Electoral Trust*. B denotes Poisson regression coefficients; SE denotes standard errors.

SM3: Question Wording

The following reflects original coding prior to recoding all variables to range from 0 to 1. Variable IDs correspond with survey question ordering. Response options and respective coding are shown in italics. For more information on the 2020 ANES, see: https://electionstudies.org/data-center/2020-time-series-study/.

Interest **(V201006)**: Some people don't pay much attention to political campaigns. How about you? Would you say that you have been very much interested, somewhat interested or not much interested in the political campaigns so far this year? (Reverse coded from ANES) *Not much interested (1); Somewhat interested (2); Very much interested (3).*

Ideology**: Liberal-Conservative Self Placement (V201200)**: We hear a lot of talk these days about liberals and conservatives. Here is a seven-point scale on which the political views that people might hold are arranged from extremely liberal to extremely conservative. Where would you place yourself on this scale, or haven't you thought much about this? *Extremely liberal (1); Liberal (2); Slightly liberal (3); Moderate; middle of the road (4); Slightly conservative (5); Conservative (6); Extremely conservative (7); Haven't thought much about this (4); Refused/don't know (omitted).*

Ideological Strength **(V201200)**: Folded measure of symbolic ideology. *Moderate (1); Slightly liberal/conservative (2); Liberal/conservative (3); Extremely liberal/conservative (4).*

Recruited: Additive index of responses to the following three questions:

1) As you know, the political parties try to talk to as many people as they can to get them to vote for their candidate. Did anyone from one of the political parties call you up or come around and talk to you about the campaign this year? [Has anyone from one of the political parties called you up or come around and talked to you about the 2020 campaign, or has no one from the political parties talked to you?] (**V202004** and **V202005**)

2) Other than someone from the two major parties, did anyone else call you up or come around and talk to you about supporting specific candidates in this last election? (**V202007**), and 3) During the campaign this year, did anyone talk to you about registering to vote or getting out to vote? (**V202008**) *No (0); Yes (1); Refused/don't know/inapplicable/no post-election data (omitted).*

Party ID **(V201231x)**: Combined measure of: Generally speaking, do you usually think of yourself as [a Democrat, a Republican / a Republican, a Democrat], and Independent, or what? *and* Would you call yourself a strong [Democrat/Republican] or a not very strong [Democrat/Republican]? *Strong Democrat (1); Not very strong Democrat (2); Independent-Democrat (3); Independent (4); Independent-Republican (5); Not very strong Republican (6); Strong Republican (7); Refused/don't know (omitted).*

Party ID Strength **(V201231x)**: Folded measure of party ID. *Independent (1); Leaning (2); Not very strong (3); Strong (4).*

Trust in Government **(V201233)**: How often can you trust the federal government in Washington to do what is right? (Reverse coded from ANES) *Never (1); Some of the time (2); About half the time (3); Most of the time (4); Always (5); Refused/don't know (omitted).*

Issues: Scaled average of responses to the following seven, seven-point questions:

1) Some people think the government should provide fewer services even in areas such as health and education in order to reduce spending. Suppose these people are at one end of a scale, at point 1. Other people feel it is important for the government to provide many more services even if it means an increase in spending. Suppose these people are at the other end, at point 7. Where would you place yourself on this scale, or haven't you thought much about this? (**V201246**)

2) Some people believe that we should spend much less money for defense. Suppose these people are at one end of a scale, at point 1. Others feel that defense spending should be greatly increased. Suppose these people are at the other end, at point 7. And,

of course, some other people have opinions somewhere in between, at points 2, 3, 4, 5 or 6. Where would you place yourself on this scale, or haven't you thought much about this? (**V201249**)

3) There is much concern about the rapid rise in medical and hospital costs. Some people feel there should be a government insurance plan which would cover all medical and hospital expenses for everyone. Suppose these people are at one end of a scale, at point 1. Others feel that all medical expenses should be paid by individuals through private insurance plans like Blue Cross or other company paid plans. Suppose these people are at the other end, at point 7. And, of course, some other people have opinions somewhere in between, at points 2, 3, 4, 5, or 6. Where would you place yourself on this scale, or haven't you thought much about this? (**V201252**)

4) Some people feel the government in Washington should see to it that every person has a job and a good standard of living. Suppose these people are at one end of a scale, at point 1. Others think the government should just let each person get ahead on their own. Suppose these people are at the other end, at point 7. And, of course, some other people have opinions somewhere in between, at points 2, 3, 4, 5, or 6. Where would you place yourself on this scale, or haven't you thought much about this? (**V201255**)

5) Some people feel that the government in Washington should make every effort to improve the social and economic position of blacks. Suppose these people are at one end of a scale, at point 1. Others feel that the government should not make any special effort to help blacks because they should help themselves. Suppose these people are at the other end, at point 7. And, of course, some other people have opinions somewhere in between, at points 2, 3, 4, 5, or 6. Where would you place yourself on this scale, or haven't you thought much about this? (**V201258**)

6) Some people think we need much tougher government regulations on business in order to protect the environment. Suppose these people are at one end of a scale, at point 1. Others think that current regulations to protect the environment are already too much

of a burden on business. Suppose these people are at the other end, at point 7. And, of course, some other people have opinions somewhere in between, at points 2,3,4,5, or 6. Where would you place yourself on this scale, or haven't you thought much about this? (**V201262**)

7) Do you favor, oppose, or neither favor nor oppose building a wall on the U.S. border with Mexico? *and* Do you [favor / oppose] that a great deal, a moderate amount, or a little? (**V201426x**; Reverse coded from ANES).

Haven't thought much about this (4); Refused/don't know (omitted).

Trump: Averaged scale of responses to the following three questions:

1) Do you approve or disapprove of the way Donald Trump has handled the coronavirus, or COVID-19, pandemic? (**V201142**; Reverse coded from ANES) *Disapprove (1); Approve (2); Refused/don't know (omitted).*

2) Do you favor, oppose, or neither favor nor oppose the U.S. House of Representatives' decision in December of last year to impeach President Trump? (**V201384**) *Favor (1); Neither favor nor oppose (2); Oppose (3); Refused/don't know(omitted).*

3) Do you favor, oppose, or neither favor nor oppose the U.S. Senate's decision in February to acquit President Trump of the impeachment charges and thus let him remain in office? (**V201387**; Reverse coded from ANES) *Oppose (1); Neither favor nor oppose (2); Favor (3); Refused/don't know(omitted).*

Abortion **(V201342x)**: Abortion rights Supreme Court. (Reverse coded from ANES) *Extremely upset (1); Moderately upset (2); A little upset (3); Neither pleased nor upset (4); A little pleased (5); Moderately pleased (6); Extremely pleased (7); Refused/don't know (omitted).*

COVID-19 **(V201392x)**: Federal government response to COVID-19. (Reverse coded from ANES) *Much too slow (1); Somewhat too slow (2); About right (3); Somewhat too quick (4); Much too quick (5); Refused/don't know (omitted).*

Religiosity **(V201433)**: How important is religion in your life? (Reverse coded

from ANES) *Not important at all (1); A little important (2); Moderately important (3); Very important (4); Extremely important (5); Refused/don't know (omitted).*

NVP: Additive scale of responses to the following 12 questions:

1) During the campaign, did you talk to any people and try to show them why they should vote for or against one of the parties or candidates? (**V202009**)

2) Did you participate in any online political meetings, rallies, speeches, fundraisers, or things like that in support of a particular candidate? (**V202013**)

3) Did you go to any political meetings, rallies, speeches, dinners, or things like that in support of a particular candidate? (**V202014**)

4) Did you wear a campaign button, put a campaign sticker on your car, or place a sign in your window or in front of your house? (**V202015**)

5) Did you do any other work for one of the parties or candidates? (**V202016**)

6) During an election year people are often asked to make a contribution to support campaigns. Did you give money to an individual candidate running for public office? (**V202017**)

7) Did you give money to a political party during this election year? (**V202019**)

8) Did you give any money to any other group that supported or opposed candidates? (**V202021**)

9) During the past 12 months, have you joined in a protest march, rally, or demonstration, or have you not done this in the past 12 months? (**V202025**)

10) During the past 12 months, have you signed a petition on the Internet or on paper about a political or social issue, or have you not done this in the past 12 months? (**V202026**)

11) During the past 12 months, have you posted a message or comment online about a political issue or campaign, or have you not done this in the past 12 months? (**V202029**)

12) During the past 12 months, have you contacted or tried to contact a member of

the U.S. Senate or U.S. House of Representatives, or have you not done this in the past 12 months? (**V202030**)

No (0); Yes (1); Refused/no post-elected data (omitted).

Income (**V202467**): Please mark the answer that includes the income of all members of your family during the past 12 months before taxes. *Under $9,999 (1); $10,000-4,999 (2); $15,000-19,999 (3); $20,000-24,999 (4); $25,000-29,999 (5); $30,000-34,999 (6); $35,000-39,999 (7); $40,000-44,999 (8); $45,000-49,999 (9); $50,000-59,999 (10); $60,000-64,999 (11); $65,000-69,999 (12); $70,000-74,999 (13); $75,000-79,999 (14); $80,000-89,999 (15); $90,000-99,999 (16); $100,000-109,999 (17); $110,000-124,999 (18); $125,000-149,999 (19); $150,000-174,999 (20); $175,000-249,999 (21); $250,000 or more (22).*

Finances (**V201503**): Now looking ahead, do you think that a year from now [you / you and your family living here] will be much better off financially, somewhat better off, about the same, somewhat worse off, or much worse off than now? *Much better off (1); Somewhat better off (2); About the same (3); Somewhat worse off (4); Much worse off (5); Refused/don't know (omitted).*

Age (**V201507x**): Respondent age. *Age in years (age in years); Age 80 or older (80); Refused (omitted).*

Education (**V201511x**): What is the highest level of school you have completed or the highest degree you have received? *Less than high school credential (1); High school credential (2); Some post-high school, no bachelor's degree (3); Bachelor's degree (4); Graduate degree (5); Refused/don't know/missing (omitted).*

Race (**V201549x**): I am going to read you a list of five race categories. You may choose one or more races. For this survey, Hispanic origin is not a race. Are you White; Black or African American; American Indian or Alaska Native; Asian; or Native Hawaiian or Other Pacific Islander? *White, non-Hispanic (1); Black, non-Hispanic (0); Hispanic (0); Asian or Native Hawaiian/other Pacific Islander, non-Hispanic alone (0); Multiple races,*

non-Hispanic (0); Refused/don't know (omitted).

***Ethnicity* (V201549x)**: I am going to read you a list of five race categories. You may choose one or more races. For this survey, Hispanic origin is not a race. Are you White; Black or African American; American Indian or Alaska Native; Asian; or Native Hawaiian or Other Pacific Islander? *White, non-Hispanic (0); Black, non-Hispanic (0); Hispanic (1); Asian or Native Hawaiian/other Pacific Islander, non-Hispanic alone (0); Multiple races, non-Hispanic (0); Refused/don't know (omitted).*

***Sex* (V201600)**: What is your sex? *Male (0); Female (1); Refused (omitted).*

Efficacy: Scaled average of responses to the following two questions:

1) For the following statements, please tell me how strongly you agree or disagree: 'Public officials don't care much what people like me think.' Do you agree strongly, agree somewhat, neither agree nor disagree, disagree somewhat, or disagree strongly with this statement? (**V202212**)

2) People like me don't have any say about what the government does.' Do you agree strongly, agree somewhat, neither agree nor disagree,disagree somewhat, or disagree strongly with this statement? (**V202213**)

Agree strongly (1); Agree somewhat (2); Neither agree nor disagree (3); Disagree somewhat (4); Disagree strongly (5); Refused/no post-election data/interview breakoff (omitted).

***Anger* (V201118)**: How angry do you feel about how things are going in the country? *Not at all (1); A little (2); Somewhat (3); Very (4); Extremely (5).*

Electoral Trust: Scaled average of responses to the following two questions:

1) In the November 2020 general election, how accurately do you think the votes will be counted? (**V201351**) *Not at all accurately (1); A little accurately (2); Moderately accurately (3); Very accurately (4); Completely accurately (5); Refused/don't know (omitted).*

2) How much do you trust the officials who oversee elections where you live? (**V201352**) *Not at all (1); A little (2); A moderate amount (3); A lot (4); A great deal*

(5); Refused/don't know (omitted).

***Trust in People* (V201237)**: Generally speaking, how often can you trust other people? (Reverse coded from ANES) *Never (1); Some of the time (2); About half the time (3); Most of the time (4); Always (5).*

SM4: Summary Statistics

	Obs	Mean	SD	Min	Max
NVP	7449	2.14	2.31	0	12
Issues	8275	3.77	1.46	1	7
Electoral Trust	8258	0.56	0.26	0	1
Trust in Government	8243	2.52	0.88	1	5
Trust in People	8261	3.19	0.90	1	5
Party ID Strength	8245	2.99	1.07	1	4
Ideological Strength	8257	2.15	1.03	1	4
Education	8149	3.39	1.11	1	5
Income	7980	11.73	6.74	1	22
Religiosity	8249	3.20	1.49	1	5
Age (in years)	7932	51.59	17.21	18	80+
Sex	8213	0.54	0.50	0	1
Race	8178	0.73	0.44	0	1
Ethnicity	8178	0.09	0.29	0	1
Recruited	7449	0.98	0.95	0	3
Interest	8279	2.39	0.70	1	3
Efficacy	7419	2.42	1.05	1	5
Anger	8263	3.58	1.20	1	5
Finances	8219	2.69	0.86	1	5

Table SM4: Summary Statistics. Table entries reflect original values prior to recoding from 0 to 1.

SM5: NVP in 2020 (with Binned *Issues*)

	(1)			(2)		
	B	SE	p-value	B	SE	p-value
Issues (Binned)	-0.13	(0.02)	0.000	-0.01	(0.04)	0.853
Electoral Trust	-0.22	(0.08)	0.010	-0.01	(0.09)	0.907
Electoral Trust x Issues (Binned)				-0.24	(0.08)	0.004
Trust in Government	0.00	(0.07)	0.988	0.00	(0.02)	0.966
Trust in People	0.29	(0.08)	0.000	0.28	(0.08)	0.001
Party ID Strength	0.13	(0.05)	0.015	0.13	(0.05)	0.018
Ideological Strength	0.64	(0.05)	0.000	0.63	(0.05)	0.000
Interest	0.91	(0.05)	0.000	0.91	(0.05)	0.000
Education	0.27	(0.06)	0.000	0.27	(0.06)	0.000
Income	0.18	(0.05)	0.002	0.16	(0.05)	0.003
Religiosity	0.02	(0.05)	0.667	0.02	(0.05)	0.644
Age	-0.11	(0.06)	0.078	-0.11	(0.06)	0.058
Sex	0.04	(0.03)	0.221	0.04	(0.03)	0.203
Race	0.13	(0.04)	0.005	0.12	(0.05)	0.012
Ethnicity	0.00	(0.07)	0.972	0.00	(0.07)	0.995
Recruited	0.62	(0.04)	0.000	0.62	(0.04)	0.000
Efficacy	0.08	(0.06)	0.203	0.08	(0.06)	0.193
Anger	0.46	(0.05)	0.000	0.45	(0.06)	0.000
Finances	-0.07	(0.09)	0.459	-0.07	(0.09)	0.420
Constant	-3.62	(0.11)	0.000	-3.70	(0.10)	0.000
N		6794			6794	

Table SM5: NVP in 2020 (with Binned *Issues*). B denotes Poisson regression coefficients; SE denotes standard errors.

SM6: Alternative 2020 *Issues*

We also tested whether other issues relevant issues (Pew 2020b) not included *Issues* (because of different measurement scales) yielded similar results. *Trump* combines responses to three questions on whether respondents favor [approve], neither favor nor oppose [neither approve or disapprove], or oppose [disapprove]: 1) the House impeachment decision for Trump, 2) the Senate acquittal decision for Trump, and 3) Trump's handling of COVID-19 ($\alpha = 0.91$). Prior to averaging these items together, we reverse coded the Senate question. *Abortion* combines responses to two questions ("Would you be pleased, upset, or neither pleased nor upset if the Supreme Court reduced abortion rights?" and "How [pleased/upset]?") with responses ranging from extremely pleased to extremely upset. *COVID-19* combines responses to two questions ("Do you think the federal government's response to the COVID-19 outbreak earlier this year was too quick, too slow, or about right?" and "Do you think it was much too [quick/slow] or somewhat too [quick/slow]?") with responses ranging from much too quick to much too slow. We recoded all of these scaled measures to range from 0 to 1.

All three measures are coded to correspond with partisan identity and ideology such that lower values represent Democratic/liberal preferences and higher values represent Republican/conservative preferences. All are strongly correlated with partisanship ($r = 0.78$, 0.54, and 0.59, respectively), ideology ($r = 0.68$, 0.59, and 0.54, respectively) and *Issues* ($r = 0.79$, 0.60, and 0.63, respectively). As shown in Table SM6, the interaction terms for these items and trust in the election are significant and in the expected direction, providing further support for the findings in our main analysis.

	(1)			(2)			(3)			(4)			(5)			(6)		
	B	SE	p-value	B	SE	p-value	B	SE	p-value	B	SE	p-value	B	SE	p-value	B	SE	p-value
Trump	-0.06	(0.04)	0.137							0.14	(0.10)	0.190						
Abortion				-0.25	(0.06)	0.000							0.04	(0.12)	0.725			
COVID-19							-0.02	(0.07)	0.828							0.37	(0.17)	0.040
Electoral Trust x Trump										-0.38	(0.17)	0.033						
Electoral Trust x Abortion													-0.58	(0.20)	0.006			
Electoral Trust x COVID-19																-0.78	(0.30)	0.012
Trust in Government	-0.03	(0.07)	0.718	-0.03	(0.07)	0.641	-0.04	(0.07)	0.620	-0.03	(0.07)	0.729	-0.04	(0.07)	0.631	-0.03	(0.07)	0.645
Trust in People	0.30	(0.07)	0.000	0.30	(0.08)	0.000	0.30	(0.07)	0.000	0.29	(0.08)	0.000	0.29	(0.08)	0.000	0.29	(0.08)	0.000
Trust in Election	-0.16	(0.08)	0.057	-0.18	(0.08)	0.027	-0.14	(0.08)	0.094	-0.01	(0.09)	0.927	0.01	(0.08)	0.877	0.01	(0.10)	0.912
Party ID Strength	0.14	(0.05)	0.014	0.13	(0.05)	0.020	0.13	(0.05)	0.014	0.13	(0.05)	0.015	0.12	(0.05)	0.025	0.13	(0.05)	0.014
Ideological Strength	0.64	(0.05)	0.000	0.65	(0.05)	0.000	0.63	(0.05)	0.000	0.64	(0.05)	0.000	0.65	(0.05)	0.000	0.63	(0.05)	0.000
Interest	0.92	(0.06)	0.000	0.92	(0.06)	0.000	0.93	(0.05)	0.000	0.91	(0.06)	0.000	0.91	(0.06)	0.000	0.92	(0.06)	0.000
Education	0.29	(0.06)	0.000	0.27	(0.06)	0.000	0.30	(0.06)	0.000	0.29	(0.06)	0.000	0.26	(0.06)	0.000	0.30	(0.06)	0.000
Income	0.16	(0.05)	0.004	0.16	(0.05)	0.005	0.16	(0.05)	0.004	0.15	(0.05)	0.006	0.14	(0.05)	0.010	0.14	(0.05)	0.006
Religiosity	-0.05	(0.05)	0.280	0.03	(0.05)	0.514	-0.07	(0.04)	0.139	-0.05	(0.05)	0.302	0.03	(0.05)	0.474	-0.06	(0.05)	0.198
Age	-0.15	(0.06)	0.015	-0.14	(0.06)	0.024	-0.16	(0.06)	0.011	-0.16	(0.06)	0.009	-0.14	(0.06)	0.020	-0.17	(0.06)	0.007
Sex	0.06	(0.04)	0.110	0.04	(0.04)	0.228	0.06	(0.04)	0.095	0.06	(0.03)	0.101	0.05	(0.04)	0.207	0.06	(0.04)	0.085
Race	0.09	(0.05)	0.062	0.10	(0.05)	0.031	0.08	(0.05)	0.128	0.08	(0.05)	0.097	0.10	(0.05)	0.046	0.07	(0.05)	0.163
Ethnicity	-0.00	(0.07)	0.967	0.00	(0.07)	0.957	-0.01	(0.07)	0.925	-0.00	(0.07)	0.975	0.00	(0.07)	0.979	-0.01	(0.07)	0.926
Recruited	0.64	(0.04)	0.000	0.63	(0.04)	0.000	0.64	(0.04)	0.000	0.64	(0.04)	0.000	0.64	(0.04)	0.000	0.64	(0.04)	0.000
Efficacy	0.12	(0.06)	0.064	0.11	(0.06)	0.094	0.13	(0.06)	0.049	0.12	(0.06)	0.061	0.11	(0.06)	0.092	0.12	(0.06)	0.052
Anger	0.54	(0.06)	0.000	0.51	(0.06)	0.000	0.56	(0.06)	0.000	0.53	(0.06)	0.000	0.50	(0.06)	0.000	0.55	(0.06)	0.000
Finances	-0.04	(0.09)	0.688	-0.05	(0.09)	0.554	-0.03	(0.09)	0.763	-0.04	(0.09)	0.657	-0.07	-0.09	0.461	-0.03	-0.09	0.738
Constant	-3.76	(0.11)	0.000	-3.68	(0.10)	0.000	-3.80	(0.10)	0.000	-3.82	(0.10)	0.000	-3.75	(0.10)	0.000	-3.85	(0.10)	0.000
N		6793			6777			6784			6793			6777			6784	

Table SM6. Alternative 2020 Issues. B denotes Poisson regression coefficients. SE denotes standard errors.
